# Getting Beyond Words With Hermann Schmitz's Idea of Felt‐Bodily Communication

**DOI:** 10.1111/nup.70080

**Published:** 2026-10-25

**Authors:** Thomas Falkenstein, Martin Nagl‐Cupal, Hanna Mayer

**Affiliations:** ^1^ Department of Nursing Science, Faculty of Social Sciences University of Vienna Vienna Austria; ^2^ Department of Nursing Science with focus on Person‐Centred Care Research, Faculty of Health Sciences Karl Landsteiner University Krems Austria

**Keywords:** embodiment, felt body, felt‐bodily communication, neo‐phenomenology, nursing

## Abstract

This article discusses German philosopher Hermann Schmitz's philosophical idea of felt‐bodily (*leibliche*) communication. Applied to nursing, his theory suggests that felt‐bodily communication with a person is the vital impetus for fostering caring relationships. Nursing often requires addressing the limitations of language, as words sometimes fail to fully express the depth of human connections. Schmitz provides a neo‐phenomenological underpinning for this theory of communication and redefines the crucial turn to the subject as being affected by another through our felt body (*Leib*). Bodily touch, such as holding hands, directly mediates the connection between felt bodies while communicating immediate meaning. In these situations, glances and touches between people convey meanings that facilitate communication beyond words, known as felt‐bodily communication. Through felt‐bodily communication, we go beyond the physical boundaries of our material body (*Körper*) to connect with other felt bodies, thereby facilitating shared meaning. This form of communication opens an atmospheric space between bodies that affects our vital drive before these moments can be translated into words. According to Schmitz, language has an evaluative aspect that enables judgement of what has been said, whereas communication occurs through the felt body before we enter the realm of language. Felt‐bodily communication bypasses such judgement; it directly mediates meaning and offers the wisdom of a relationship in which care thrives beyond words.

## 1 Introduction

In the past, both grand and middle‐range theories of care have focused on the encounter: care as a relational state of being. The immediate relational nature of human existence is paramount to caring and serves as a communicative and transformative act. Key scholars—including Wiedenbach (1964), Watson (1979), Leininger (1984), Benner and Wrubel (1989), Swanson (1991), and Boykin and Schoenhofer (1993)—have shaped this perspective, which continues to inform contemporary discourse. Their body of work has contributed to a nuanced understanding of caring and suggests that the essence of every encounter is the relational dimension, thereby reflecting the nature of care.

These theories allow us to see the encounter as integral to caring, transcending verbal exchanges and encompassing the non‐verbal dimension of the relationship. They further emphasise that relationality is fundamental to the caring process. The unspoken and the space in between forge a connection through personal presence in caring; as joy and suffering flow through our bodies during encounters, caring becomes a powerful act of bridging. Everyone involved in the relational act of care participates in transformation within this shared relational space, shaped by presence and actualising their wholeness through the encounter and the reciprocal moment of seeing.

In another context, Carper (1978) outlines four patterns of knowing in nursing, which articulate the relationality of caring and provide insight into the nature of nursing knowledge. These patterns highlight the ways in which nurses bring subjectivity back into the world of objectivity; aesthetic knowing, a distinct and holistic form of knowledge, may integrate empirical understanding and personal experience, and it plays a significant role in encounters based on the ethical dimension of care (Carper 1978). This unique and holistic nature of nursing knowledge emphasises the depth and complexity of the discipline. Likewise, it underscores nursing as a transformative process of understanding, shaped by the communicative aspects of care that move beyond mere objectivity and recognise human beings as having the capacity to communicate. This is a relational process, highlighting the inherent mutuality in nursing (Fleischer et al. 2009). Meleis (2018) adds to this perspective by noting that communication, in the discipline of nursing, is about relationships and interactions that give voice to individuals and empower them to express themselves. Following this logic, Bender (2018) suggests that nursing cannot be conceived separately from an ontological metaparadigm; to be a nurse is to be engaged in a relationship based on the relationality of care, which begins with recognising one another as interdependent partners in the nursing process (Bender 2018). Furthermore, communication in nursing involves more than technical skills, as such skills fail to capture the holistic nature and complexity of nursing relationships (Mayer and Wallner 2024). For nursing, communication is not merely a tool; it is anchored in the ontological basis through which we find one another. The starting point for how an encounter takes place and unfolds as a shared, collaborative process is the other's aliveness. Communication is an essential part of nursing; it is the core and art of nursing and the key to building professional relationships.

This notion of the holistic nature of communication as a shared process is consistent with the concept of felt‐bodily (*leibliche*) communication described by Hermann Schmitz (1928–2021), which is expressed through the act of sharing in our nature of being. In their review of nursing theories, Moers and Uzarewicz (2012) identify the ongoing need to overcome the threat of objectification in care. This collective responsibility requires moving beyond the technical mechanisms of standardisation in nursing and embracing a way of being that recognises our existence as the felt body (*Leib*) in Schmitz's sense of affective involvement. Acknowledging felt‐bodily communication highlights the potential of the relational dimension in everyday care—a moment that goes beyond objectifying actions and reveals a way of being in relation. For Schmitz (2010, 2016b), the apparent boundaries between self and others dissolve in such situations, and a dialogue develops that does not rely on language.

With this in mind, I take a closer look at felt‐bodily communication, which enables relationships through direct encounters beyond words, demonstrating its relevance to nursing. This essay introduces the concept of the felt body in Section 2, provides an overview of Schmitz's neo‐phenomenological foundations in Section 3, explores the idea of felt‐bodily communication beyond words in Section 4, discusses its role in nursing in Section 5, and concludes with a reflection on its meaning.

## 2 The Concept of the Felt Body

Before I move beyond words, I briefly review the bedrock for understanding the felt body. The following sections emphasise the meaning of the felt body, which extends beyond the material body (*Körper*):

Through the two words ‘Leib’ (the felt body) and ‘Körper’ (the material body), it is easy for us Germans to distinguish the felt body from [the] visible (and otherwise perceptible) material body. Other languages need to resort to cumbersome paraphrases. It takes the felt body [Leib] to be the epitome of everything that someone can feel of themselves (as belonging to themselves) in the vicinity (not always in the bounds) of their material body without drawing on their five senses and perceptual body scheme, i. e. the habitual image of one's own body gained through experience (in particular vision and touch).

(Schmitz 2017, 9)

The non‐material bodily dimension of nursing becomes apparent in its significant role in everyday life. However, daily care involves much more than physical and body‐related activities; it always entails a felt‐bodily dimension. Whether we seek an encounter or wish to avoid it, the aesthetic aspect of the felt‐bodily dimension in nursing is always present. While knowledge related to the material body—such as anatomy, physiology, and pathology—is crucial for nursing practice, the felt‐bodily dimension imbues each action with an aesthetic quality. Moers and Uzarewicz (2012, 137) highlight this aspect in relation to personal hygiene. Even in seemingly purely physical interactions, felt‐bodily aspects such as touch are essential; personal hygiene is not merely a functional measure.

Schmitz's concept of the felt body is more than a theoretical notion; it is a profound understanding that underpins relationships. This concept reveals a dynamic beyond the material body by building a bridge to the body's pre‐dimensionality. Thus, the felt‐bodily dimension plays a vital role in any human relationship (Schmitz 2015).

For Schmitz (2015), the felt body has no skin or surfaces that belong to the material body; its location is absolute, and it is directly experienced as a pre‐dimensional volume. Schmitz emphasises that the scientific concept of the body tends not to differentiate between the animate and the inanimate. In contrast, the felt body describes a relational experience of oneself and the world that extends beyond purely scientific interpretations. Human existence is thus perceived through affective involvement, with the felt body contributing to an essential dimension of being.

Affective involvement between the *ego* and the *alter ego* opens up an understanding of the turn from the material body to the felt body. Early philosophical discussions traditionally associated the material body with the objective and the felt body with the subjective. The following section briefly explains the felt body. The aim is to reframe the terminology of consciousness, which has long been of philosophical interest and is essential for understanding what we feel in our affective involvement with other human beings.

Phenomenology, as advanced by Edmund Husserl (1859–1938), was dedicated to the things themselves, revealed in the consciousness of something. His *epochē* and eidetic reduction are methods that shift the focus from questions of existence to the essence of the phenomenon (Schülein and Reitze 2021). Regarding the terminology of the material body and the felt body, Fuchs (2018, 43–44) underlines this idea of distinction with a compelling example from Husserl's discussion of the problem of double sensation: specifically, when one hand touches the other, the hand that is touching becomes the hand being touched. Consequently, two distinct modes of access to the body emerge simultaneously. This example demonstrates that the material body and the felt body are not strict opposites; they are complementary dimensions of human perception. Fuchs (2018) adds that, while the felt body is not central to Husserl's phenomenology, it is repeatedly thematised in his analyses of consciousness. Consciousness experiences the felt body, not through reason but through perception grounded in one's felt‐bodily sensation.This is how the felt‐bodily experience of the subject arises, which is inherently one's own. Martin Heidegger (1889–1976) broadened the horizon by shifting the focus from consciousness to *Dasein*, within which phenomena appear and are grasped in their relational structure. In Heidegger's existential analysis, this concept acquires an ontological dimension, as *Dasein* emerges as a situated being‐in‐the‐world (*In‐der‐Welt‐sein*). Influenced by Husserl's transcendental phenomenology, Maurice Merleau‐Ponty (1908–1961) bridges this gap by focusing on pre‐reflective, felt‐bodily experience—an immediate and unanalysed awareness of the felt body and its sensations, as the foundation of existence. However, Merleau‐Ponty's work leaves the affective involvement of the felt body relatively underexplored.

Schmitz's contributions play a significant role in contemporary phenomenological debates and enrich the understanding of the interplay and mediation between subjectivity and objectivity. According to Fuchs (2018), Schmitz argues that subjectivity remains deeply bound to lived experience. It does not merely denote something but rather emerges from felt‐bodily experience. In this case, affective involvement is at the core of the subjectivity represented by the felt body. Schmitz places these implicit aspects at the centre of the felt‐bodily experience and brings them into the world as an overarching dimension of shared being (Fuchs 2018). His remarks reflect the discourse on the objective and subjective body in an attempt to overcome the dogmatic either‐or dichotomy.

## 3 Neo‐Phenomenological Foundations

Neo‐phenomenology, as outlined by Schmitz (2003), provides a philosophical foundation and a theoretical framework for communication. Building on this, Uzarewicz and Uzarewicz (2005) emphasise the importance of felt‐bodily involvement in care. Schmitz (2003) asserts that phenomena reflect the world and are disclosed through affective experiences. As Schmitz et al. (2011, 243) note, “A phenomenon for someone at a time is a state of affairs of which the person in question cannot in earnest deny that it is a fact.” According to Schmitz (2007, 5, 116), the philosophy of neo‐phenomenology represents humans’ self‐reflection on finding themselves in their surroundings—which is not a secluded province but a universal sounding board that may act as an amplifier of the felt body. For Uzarewicz and Uzarewicz (2005), this resonance is omnipresent in relationships established by professional caregivers; the felt body is an essential component of care, therapeutic measures, and social support, making it indispensable for the caring dimension of nursing.

Schmitz's phenomenological approach draws attention to the immediacy of involuntary life experiences that affect us directly and place the felt body in the foreground. The way we think is based on an abstraction that acts as a separating filter through which theories and judgements flow. Every culture has created abstractions, such as language types and historical imprints. Neo‐phenomenology involves allowing the involuntary experiences of life to speak again without falling entirely into the aberrations of language (Schmitz 2021, 48). Through this philosophical approach, phenomena are no longer treated with apodictic certainty or reduced to singular interpretations. Instead, the focus shifts decisively to the subject. As part of neo‐phenomenology, felt‐bodily communication goes beyond the material body and encompasses social living spaces and their atmospheric dimensions (Schmitz 2016a, 12).

For Schmitz (2021), the psychologistic–reductionist–introjectionist objectification, in which the world breaks down for the human being into an external world and an internal world to which experience is confined, ultimately results in a predominance of reductionism through a fixation on intellectuality at the expense of bodily perception. Rejecting the view that subject and object are comparable to solid bodies in a causal relationship, he emphasises the entanglement of the material body and the felt body (Schmitz 2021, 49). According to Schmitz's philosophy, the felt body pre‐reflectively interacts with its environment (Schmitz 2007, 5). This guiding principle introduces an essential tenet of neo‐phenomenology, underscoring the originality and uniqueness of the subjective while offering a resounding critique of the prevailing canon of objective veridicality (Schmitz 2007, 2016a). Schmitz (2015, 28–30) critiques the traditional dogma of physiologism, which breaks down the world for every human being into an external and an internal world. Accordingly, information from the external world first reaches the internal world through the transport and transformation of physical signals via the sensory organs and the nervous system. Motor reactions directed towards this external world are conveyed by the reverse export through neural channels. This approach proves untenable, as there is no time for such a complicated sequence of intellectual processes. In criticising the import–export relationship—in the sense of a psychologistic–reductionist–introjectionist way of thinking—Schmitz rejects placing the perception of the material body on the same level as that of the felt body.

## 4 Communication Beyond Words—Felt‐Bodily Communication

Moving beyond a theoretical view of communication as a collection of attributes, it becomes clear that it reflects neither purely objective nor purely subjective states of affairs. Confining it to a single category would not capture the complexity of the communicative moment. In this section, I explore felt‐bodily communication as an essential expression that generates communicative impulses within the dynamic between two human beings. However, this description does not cover the full spectrum of Hermann Schmitz's concept of the felt body in its neo‐phenomenological foundations, since the focus is explicitly on the immediacy and power of interpersonal relationships in felt‐bodily communication beyond words. Schmitz's (2011, 2015) theory of felt‐bodily communication describes a dynamic and engaging phenomenological framework that encompasses human interactions beyond solitude. Felt‐bodily communication is in constant motion; incorporation (*Einleibung*) acts as a bridge within the vital drive, while excorporation (*Ausleibung*) loses itself in a gaze into the depth of space and proceeds into expandedness. In the case of antagonistic incorporation, two distinct relational dynamics emerge: partners are either bound in a one‐sided distribution of roles (one‐sided antagonistic incorporation, *einseitige Einleibung*) or they enter into a dialogue concerning their changing distribution of roles (mutual antagonistic incorporation, *wechselseitige Einleibung*)—a dance between the contractedness and expandedness of felt‐bodily communication that goes beyond the realm of words.

According to Schmitz (2011, 2015), felt‐bodily communication between two people is not a one‐way but, rather, a two‐way channel of communication that exists beyond words. This interwoven aspect between human beings, which I refer to as a particular felt‐bodily mode of communication, is especially significant when considering the non‐linguistic moments we can bridge through felt‐bodily channels. Schmitz describes the mutual incorporation of the dynamically fluctuating dominant role in the shared vital drive between two people. It corresponds to the rhythmic change of dominance between tension and swelling in the vital drive of the individual. In this process, dominance is tossed back and forth like a ball at short intervals. An important vehicle in this interplay is the exchange of glances, for example, during a conversation. In turn, one‐sided incorporation is rigid in both role distribution and dominance. The passive (incorporated) partner is bound to and focused on the dominant one, regardless of whether or not the latter turns towards the former. This creates a situation in which a gaze can capture us, that is, hypnotise us. For example, we may find ourselves entirely captivated and fascinated by that gaze. This intense, virtually hypnotic effect is an essential aspect of this form of communication, which must be considered (Schmitz 2011, 39–41).

According to Schmitz (1989, 2008), felt‐bodily communication—a form of interaction deeply tied to the concept of expression—represents the moment of direct encounter: the intense fascination that arises between the *ego* and the *alter ego*. This intense fascination, as Schmitz (1989, 31–32) describes it, is a deep and irresistible captivation that arises when a person is so affected by something that they fall under its spell and are inevitably driven to follow it. In such cases, these forces affect and afflict the person, shaping their state of well‐being and their behaviour, both in suffering and in response.

Schmitz relates felt‐bodily communication to speechless thinking, expressed through immediacy, as intelligence beyond words (Schmitz 2010, 2016b). Linguistic rules take a secondary role in the expression of felt‐bodily communication. Schmitz (2010, 86) describes the form of intelligent thinking as the handling of meanings—comprising states of affairs, programmes, and problems— that is, the ability to achieve success not by mere application of pre‐established schemata, but by reinventions adapted to each situation. Thinking with felt‐bodily intelligence grasps, reshapes, and answers situations; it is not teleological in this way because, lacking linguistic explication, it possesses no single programme. However, it can be measured by its success (Schmitz 2010, 94).

Whether or not we speak, communication is possible if we find a way in which shared understanding can emerge. Felt‐bodily communication presents itself as a relational movement in which we connect and communicate through our felt bodies. The key is to look beyond the traditional channels of communication. It is about thinking beyond the existing functions of language and defining its success in terms of a shared moment: bringing a smile to someone's face, moving one's (material) body to the joy of music, and creating a moment together that unites us in the world.

Felt‐bodily communication may be found in the pre‐reflective and pre‐symbolic realm, grounded in an immediate, non‐linguistic understanding which encompasses the situation and participants’ interaction in an encounter. Kettner (2007) describes how, in terms of felt‐bodily communication, it is helpful to reflect on what the felt body means and distinguish it from the usual division between verbal and pre‐verbal communication. He discusses how felt‐bodily communication is complementary in a unique and personal way, providing the missing link between subjective and objective, going beyond the old understanding of two separate worlds. The abstraction of felt bodies relies on cognitive understanding, with the danger of losing the involuntary experience of life encountered in the immediacy of communication beyond words.

Neo‐phenomenology is evident in felt‐bodily communication, which fosters a more profound understanding through resonance with our felt‐bodily counterpart whilst preserving the intimacy of the moment without relying on language. In this way, communication becomes a direct expression that transcends words and gives form to the caregiving relationship. The following passage provides insight into the relationship‐building process that unfolded during the writing of my dissertation. First and foremost, the focus is on antagonistic incorporation in felt‐bodily communication between parents and their children with care needs that extends beyond words. I connect the experience of my first encounter with the parent and her child in their home with the neo‐phenomenological understanding described by Schmitz. In what follows, I use this narrative to illustrate how I, as a researcher, established a relationship.

Felt‐bodily communication is more than a way of understanding communication theoretically; it goes beyond surface‐level impressions to engage as a person in a shared moment. Engaging with the person as a felt‐bodily movement makes dialogue beyond words possible. At this point, I want to describe my visit with a child who served as a teacher of communication, in which our communion went far beyond the bodily moment. Sanja's mother offered me the opportunity to get to know her child and her way of communicating. As part of my dissertation, I spoke with parents about communicating with their children requiring care. Here, I describe my first meeting with Sanja in the company of Jasmina, her mother, at home:

When **I met Sanja** and her mother, Jasmina, for the first time at the family home, it was a few days after Sanja's birthday. **Sanja greeted me in her room with a happy smile**, and we began the process of getting to know each other. I could feel our gazes slowly searching for one another. Her gaze followed mine, and her bright smile told me I was welcome. I took my time and slowly approached Sanja, all the while feeling the tension in her body. She turned to me and did not avert her gaze. She looked at her mother for a moment, as if searching for confirmation, before refocusing on my movements. I approached her step by step. Her expression was open and curious, sincere, unprejudiced, and simply honest; the intensity we shared was palpable in our exchange. My intonation, the rise and fall of my voice, revealed something of my personal involvement as I felt compelled to introduce myself and explain the reason for my visit. Sanja remained attentive and held my focus, giving me her full attention. Slowly, we came closer; our different communication channels merged into one. I was then permitted to take her hand and build a bond with her through touch (Figure 1). Finally, we listened to music, as Jasmina shared her child's favourite tunes. The fast, rhythmic, stirring music brought us closer and into a shared atmosphere of ease and comfort.

**Figure 1:**
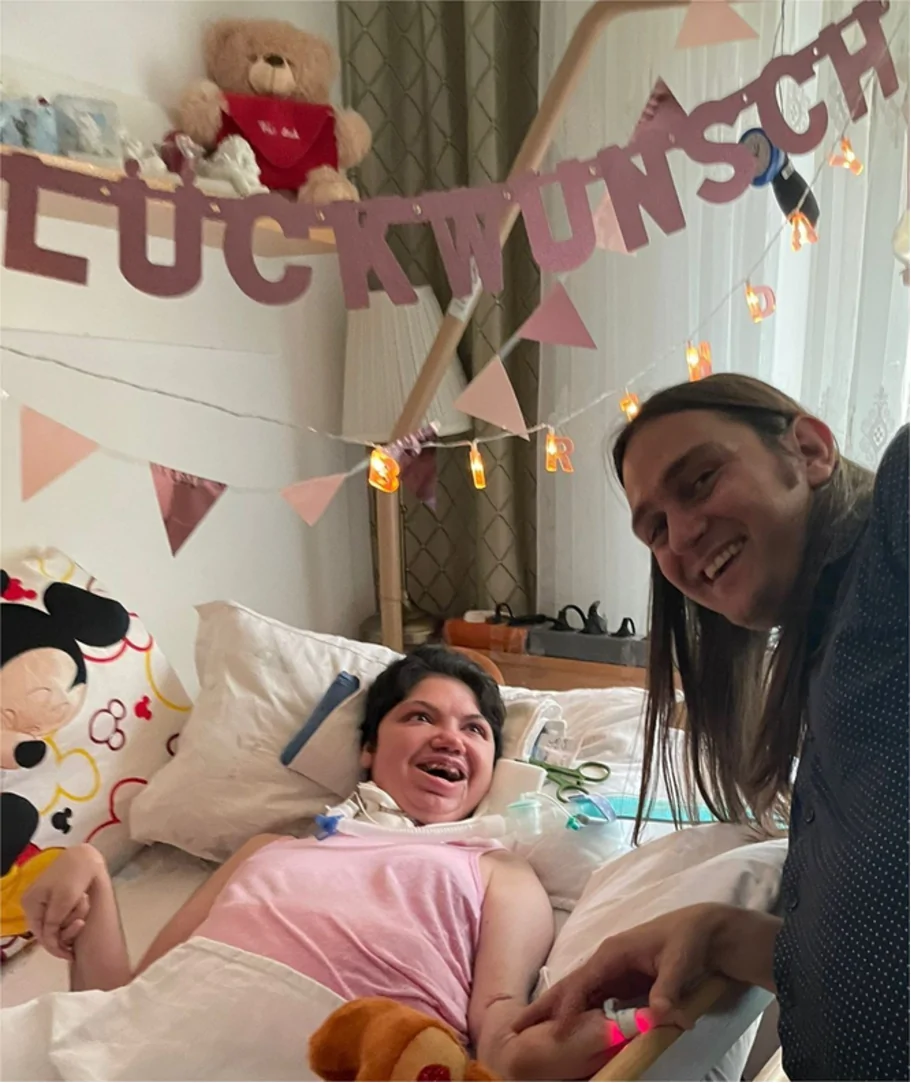
Sanja and me.

**The movement of our gaze carries an overarching resonance.** Schmitz (2011, 2015) points out that seeing leads to communication with felt‐bodily movement patterns that affect us and reveal their direction; we absorb the other person's movement, approach this figure, and grasp its character even before thinking about it. The expression of a movement is perceived through the gaze and becomes sensually and intuitively tangible to us. In this way, movements are communicated felt‐bodily, that is, passed on and reciprocated as in a dialogue beyond words. Sanja and I interact as partners; our gazes find harmony in my movements; her eyes follow me and form a dialogue that involves me as if in a dance in which I resonate with her. **Sound becomes the echo of being.** Schmitz (2011, 2015) argues that listening makes us feel the closeness of another person's voice as it approaches us. We let ourselves be carried away by the movement of what we hear and follow its form as it unfolds in expression. For both Sanja and me, her voice and my voice can also be part of such unfolding beyond verbal patterns—sometimes a mere laugh—which reveals something about the situation. At once, it shows and accompanies the action in its uniqueness. Thus, we are brought together in that shared moment. **Touch conveys an intimate moment of presence.** Schmitz (2011, 2015) emphasises touch as a channel beyond the surface of the material body—a skinless felt body with no surface. The boundaries of the material body are extended inward through touch. Touch is not merely a sense but a quality that surpasses the senses and represents a profound relational dimension. It is straightforward and involves a deep level of felt‐bodily communication. Being touched is an essential, highly intense part of our interaction; a mere touch leads to immersion and an immediate, authentic connection with the other.

Within felt‐bodily communication between counterparts, the suggestion of motion, which is also called the course of *gestalt*, unfolds beyond any single sensory dimension. It is not seeing, but the gaze that mediates between our felt bodies and exerts an atmospheric, compelling, binding, and suggestive force. The gaze can move around and turn inwards (Schmitz 2015, 33, 40). This suggestion plays a key role in understanding perception, as it builds a bridge between the felt‐bodily state and what is perceived (and vice versa). With this understanding, we feel the other's movement directly and can fully encounter them (Schmitz 2011).

Synaesthetic characters refer to intermodal qualities distributed across different senses, which provide an overarching dimension (Schmitz 2011, 33); Schmitz notes, for example, warm colours and bright tones (Schmitz 2021, 41). These qualities often, though not always, carry the names of specific sensory qualities (Schmitz 2011, 33) and open channels of felt‐bodily communication. Together with the suggestion of motion, these bridging qualities constitute an overarching quality that affects felt bodies and serves as a bridge within the vital drive where felt‐bodily communication begins (Schmitz 2011, 2015). This creates an atmosphere in which the closeness of the situation takes place, and in which Sanja and I have arrived. Indeed, music can have an additional, overarching theme; in this case, as Schmitz (2011, 2015) argues, it has a quality of solidary incorporation (*solidarische Einleibung*) that emerges whenever we experience a shared theme: fast, rhythmic, stirring music conveys a shared, overarching atmosphere, bringing us closer and into a joint situation.

According to Schmitz (2011, 2015), our senses—such as seeing, hearing, touching—connect their physical properties through bridging qualities to form felt‐bodily tangible channels. These felt‐bodily channels allow communication without consciously using a particular sensory organ or attributing specific properties to it. In this context, Schmitz does not discuss classical sensory perception, which refers to the traditional understanding of how our senses work (Schmitz 2011, 2015): these channels serve as media of communication on a felt‐bodily level; that is, with these media, one may touch people in their innermost being (Uzarewicz and Uzarewicz 2022, 605).

We are deeply affected by the meaningful impressions conveyed through the channels of felt‐bodily communication within our felt bodies. Felt‐bodily communication goes beyond boundaries and is not limited by spatial or dimensional constraints. In this way, we build bridges for one another, which also means being affectively involved. Therefore, our bodies are not closed but resonant systems that unfold immediately in the moment of encounter through the channels of felt‐bodily communication. The immediacy of communication is revealed in this process, as it does not adhere to linguistic patterns. In reciprocal dialogue, shared evidence can emerge. A sense of community can arise from this resonance as you‐evidence—a felt‐bodily bond (Schmitz 2015). Both felt‐bodily partners maintain this relationship as a unified whole, as Schmitz (2017) demonstrates below:

As a matter of fact, you‐evidence does not result from a projection of myself into the other, but from a form of involvement in the sense that something is done to me. i. e. by entanglement in the joint drive of reciprocal incorporation in which the partners confront one another with the same armor of tension and swelling.

(Schmitz 2017, 17)

Openness to the interlocking, overlapping channels of felt‐bodily communication describes what connects us beyond words, what realises the overarching power of our sensory qualities. We need to clarify how we deal with what touches us repeatedly, what touches us involuntarily and becomes part of our world. How do we transform our environment without the risk of dividing our world into an inside and an outside? In care, we use the form of aesthetics to give tangible expression to what is revealed between us; when we enter into dialogue, this means going back to the source of felt‐bodily communication before translating it into a language that reduces the situation to a snapshot of our world. While necessary for our abstraction of understanding, this reduction should be made cautiously to avoid misinterpretation.

## 5 Reframing Communication: Co‐Production Towards an Art of Nursing

Communication has its own dynamics that unfold in shared presence as a relational process through which mutual understanding emerges between people. The prefix com*‐*, as used in communication, indicates that it is not about a single mode of understanding. It points to the communal dimension of what is communicated from person to person. Mayer and Wallner (2024) locate this within the complexity of nursing practice, which is driven by co‐production. Swanson (1991, 1993) illuminates the multiplicity of shared communication in her caring theory, particularly when multiple ways of knowing emerge in situations in which caring entails commitment and responsibility for what matters to the person. As Swanson notes, nursing practice involves “*blended understandings of the empirical, aesthetic, ethical and intuitive aspects of a given clinical situation and a nexus of maintaining belief in, knowing, being with, doing for and enabling the other*” (Swanson 1993, 357).

Nursing unfolds as a meaningful communicative process: an interplay of expression and impression in each unique situation. Building on this view, Chinn et al. (2022) anchor embodiment within aesthetic knowing. Their account of embodiment can be linked to felt‐bodily communication through its emphasis on the capacity to resonate with the atmospheres that emerge in a shared caring situation. Boykin and Schoenhofer ([1993] 2013; 2000), in their theory of nursing as caring, emphasise the relational element of synchronising with the whole person as the focus of care. Such synchronisation represents the central element of a caring relationship that encompasses the entire nursing situation and manifests the connection between nurses and care recipients in the moment, thereby enhancing personhood: “*Thus, we understand [the] nursing situation as a shared lived experience in which the caring between nurse and nursed enhances personhood.* The nursing situation is the locus of all that is known and done in nursing” (Boykin and Schoenhofer [1993] 2013, 13; emphasis in original). Attending to this exchange reflects the art of nursing, specifically when nurses maintain person‐centredness in line with the framework of McCance and McCormack (2016). They suggest that being sympathetically present requires well‐developed interpersonal skills, namely ways to communicate with the person “dependent on the people” and “based on what we know about them as people” (McCance and McCormack 2016, 44). Being sympathetically present acts as a phenomenological process which attends to the whole and engages each person's ways of being (McCance and McCormack 2016; for empirical evidence, see McCance et al. 2021). An aesthetic knowing of the entire situation may be essential to the dynamic of the caring process.

Communication reflects a hermeneutical stance (Figure 2), balancing professional knowledge (objective facts) with attention to the person involved in the caring process (subjective facts) and offering companionship through the complexities of the health system. The art of nursing sustains the heart of the situation as a co‐production, in which nurses apply their professional knowledge, grounded in practice and conveyed through shared terminology. According to Schmitz (2016a), such meaning is objective if anyone can express it (not just name it), provided they know enough and can speak well enough (10). As a prelude to explicating the situation, communication entails a rational grasp of the situation and attentiveness to channels of felt‐bodily interaction, thereby supporting a hermeneutic practice that emphasises a reflective stance attuned to felt bodies, particularly when communication extends beyond words (Moers 2012; Moers and Uzarewicz 2012); this process begins with being affected in one's way of being there.

**Figure 2:**
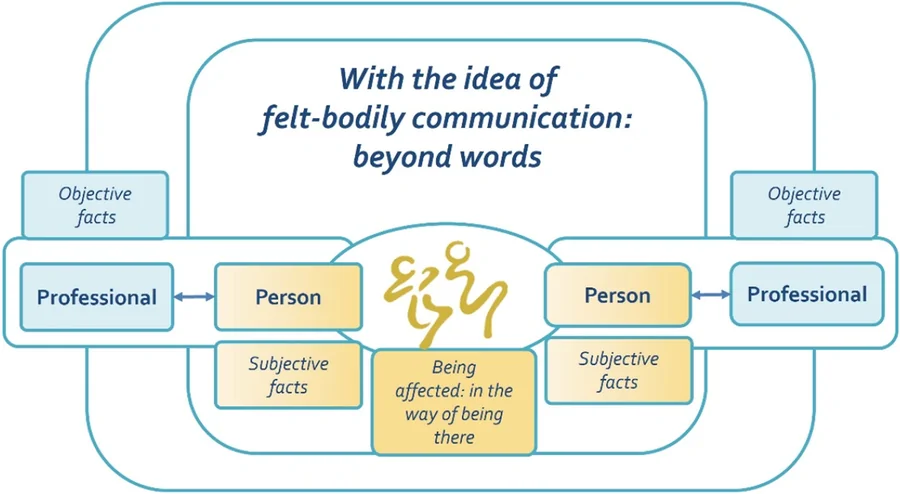
The idea of felt‐bodily communication beyond words.

In the moment, the atmosphere unfolds through a fluid hermeneutic mode of thinking alongside analytical intelligence. Hermeneutics is a way of thinking that carefully draws meaning from a situation while maintaining its wholeness and integrity. In this way, analytical thinking addresses the challenging elements of the situation (Schmitz 2010, 86–87), underpinned by nurses' professionalism and affective‐moral involvement; nursing is a co‐produced process with a reflective stance. Drawing on Schmitz, felt‑bodily intelligence emerges through channels of felt‑bodily communication and shapes the atmosphere of the moment (Schmitz 2010). In this dynamic, nurses engage their own felt bodies by attending to the subjective facts. Schmitz (2016a) notes that meaning is subjective if only one person can express it on their own behalf; this is the case when the meaning deeply affects them (10). Felt‐bodily communication may disclose moments beyond words within nursing's mutual communicative dynamic, drawing on Schmitz's (2007, 5) notion of reflective moment of finding oneself in one's surroundings. Laver illustrates this through the uniquely challenging personal experience of silence, which can be fruitful for self‐recollection as a person and a professional: “Returning to silence is to return to a constant process of professional transformation that can enable ways of knowing and being” (Laver 2024, 8).

Nursing involves balancing objective facts with subjective facts; it addresses the tension between professional understanding and personal commitment without belonging wholly to either world. To be in a caring arrangement means to relinquish the traditional conception of contact with the person and instead engage with and become familiar with both worlds. Language patterns may narrow a situation to a snapshot of reality. Schmitz (2011, 2016b) implies that language is constructed, structured, and logically processed; it remains indispensable for representing and communicating aspects of reality. At the same time, it cannot fully capture the complexity of the lived expression of felt bodies (Schmitz 2011); in such a narrowing, there is a risk of losing their situational expression. From this perspective, one can argue for linking the action and attitude of felt‐bodily communication with language. In this way, nursing requires continuous movement between these worlds, suspending dimensionality to preserve the subject's integrity.

A caring relationship can arise in both professional and informal encounters. In each case, it grows out of care for the other and begins with being there for the person. Felt‐bodily communication can open a situation in which people connect deeply. Opening entirely to the situation means actively listening to the other person, guided not by words alone but by what matters in the moment. For Sanja and me, this is how we listen otherwise; each encounter offers an opportunity to enter a shared process. Therefore, it is helpful to explore the dimensions of communication and allow them to unfold. We attend to the relational moment, for example, a favourite piece of music that Sanja shares with me in that moment, to find a common theme that accompanies us in communication, an element of solidarity that can anchor us, and to which we can return.

## 6 Conclusion

Nurses play a critical role in navigating the process of felt‐bodily communication. While verbal modes of expression can be valuable for understanding what affects us, they are always tied to a level of abstraction in a relational context. The perception of the relational context and the engagement with interpersonal relationships are of vital importance for them. It is a holistic endeavour, not just a cognitive act, as Schmitz (2010) illustrates through his concept of felt‐bodily intelligence.

Building on this premise, relationship‐building is not reduced to, but requires, interpersonal trust, especially for the felt body, which goes beyond formal and informal contact points. The quality of the relationship is what fosters felt‐bodily communication. It is a responsibility within nursing practice to create these supportive spaces, empowering nurses to reach people in the moment of encounter (Uzarewicz and Uzarewicz 2005). I have attempted to demonstrate that communication, especially in nursing, is not a one‐sided process limited to verbal patterns. The dimensions and level of felt‐bodily communication in care go far beyond this process. The underlying felt‐bodily background is a mediating element in the communication path that balances the depth of the relationship and transcends spoken language. It means meeting at eye level and respecting the expression of the other rather than assuming a position of superiority and overlooking how the other communicates. The art and heart of nursing lie in going beyond the traditional and instead finding ways of personal being, perceiving expressions beyond the structural objective of the world, and paying heed to the subjective facts of the world. This holistic nature of human communication highlights both the complexity of relationships and the depth of nursing practice.

## Ethics Statement

All data are part of a PhD dissertation and were obtained with the approval of the relevant Ethics Committee (University of Vienna, Austria, reference number 01079).

## Consent

All data were collected with the participants’ consent.

## Conflicts of Interest

The authors declare no conflicts of interest.

## Declaration of Generative AI in Scientific Writing

The first author (TF) used Grammarly and DeepL (including DeepL Write) for the language‐editing process. Additionally, tools such as ChatGPT (OpenAI) were used to improve readability. The entire content was written solely by the human authors. At the end of the process, a human editor completed the final revision.

## Data Availability

The data are part of a PhD dissertation and are available exclusively to the first author.
